# Time-resolved scRNA-seq reveals transcription dynamics of polarized macrophages with influenza A virus infection and antigen presentation to T cells

**DOI:** 10.1080/22221751.2024.2387450

**Published:** 2024-08-12

**Authors:** Jiapei Yu, Congcong Shang, Xiaoyan Deng, Ju Jia, Xiao Shang, Zeyi Wang, Ying Zheng, Rongling Zhang, Yeming Wang, Hui Zhang, Hongyu Liu, William J. Liu, Hui Li, Bin Cao

**Affiliations:** a Department of Pulmonary and Critical Care Medicine, National Center for Respiratory Medicine, National Clinical Research Center for Respiratory Diseases, Center of Respiratory Medicine, China–Japan Friendship Hospital, Beijing, People’s Republic of China; b Institute of Respiratory Medicine, Chinese Academy of Medical Sciences and Peking Union Medical College, Beijing, People’s Republic of China; c Graduate School of Peking Union Medical College, Chinese Academy of Medical Sciences, Peking Union Medical College, Beijing, People’s Republic of China; d THU-PKU Joint Center for Life Sciences, Tsinghua University, Beijing, People’s Republic of China; e Department of Infectious Disease, Beijing Friendship Hospital, Capital Medical University, Beijing, People’s Republic of China; f Department of Pulmonary and Critical Care Medicine, Clinical Center for Pulmonary Infections, Capital Medical University, Beijing, People’s Republic of China; g National Institute for Viral Disease Control and Prevention, Chinese Center for Disease Control and Prevention, Beijing, People’s Republic of China

**Keywords:** Influenza A virus, time-resolved, polarized macrophages, antigen presentation, specific T cell response

## Abstract

Throughout history, the influenza A virus has caused numerous devastating global pandemics. Macrophages, as pivotal innate immune cells, exhibit a wide range of immune functions characterized by distinct polarization states, reflecting their intricate heterogeneity. In this study, we employed the time-resolved single-cell sequencing technique coupled with metabolic RNA labelling to elucidate the dynamic transcriptional changes in distinct polarized states of bone marrow-derived macrophages (BMDMs) upon infection with the influenza A virus. Our approach not only captures the temporal dimension of transcriptional activity, which is lacking in conventional scRNA-seq methods, but also reveals that M2-polarized *Arg1*_macrophage cluster is the sole state supporting successful replication of influenza A virus. Furthermore, we identified distinct antigen presentation capabilities to CD4**^+^** T and CD8**^+^** T cells across diverse polarized states of macrophages. Notably, the M1 phenotype, exhibited by (BMDMs) and murine alveolar macrophages (AMs), demonstrated superior conventional and cross-presentation abilities for exogenous antigens, with a particular emphasis on cross-presentation capacity. Additionally, as CD8**^+^** T cell differentiation progressed, M1 polarization exhibited an enhanced capacity for cross-presentation. All three phenotypes of BMDMs, including M1, demonstrated robust presentation to CD4**^+^** regulatory T cells, while displaying limited ability to present to naive CD4^+^ T cells. These findings offer novel insights into the immunological regulatory mechanisms governing distinct polarized states of macrophages, particularly their roles in restricting the replication of influenza A virus and modulating antigen-specific T cell responses through innate immunity.

## Introduction

Influenza A virus infection can cause severe respiratory symptoms, and its high transmissibility has historically had significant adverse effects on human health and the socio-economic environment [1]. For example, recent studies have shown that PB1-F2 can induce affective disorders by inhibiting dentate gyrus (DG) oligodendrocyte development and regulating synaptic plasticity after IAV infection [2]. Despite unremitting efforts to prevent and control the spread of influenza A virus, vaccines face challenges due to the constant drift and transfer of antigens, while antiviral drugs also face resistance issues [3]. Macrophages are a kind of innate immune cells that exist in the lungs abundantly [4]. They have a strong ability to phagocytosis, inhibit infection, clear apoptotic cells, and are the main defense against the invasion of influenza A virus.

Macrophages can undergo polarization into distinct states in various microenvironments, a process crucial for their immune regulation. Each state of macrophage polarization is reversible and serves as an essential mechanism for macrophages to adapt to changes in the surrounding microenvironment. Two main extreme types of macrophages were classified as M1 (classically activated type) and M2 (alternatively activated type) [5]. Polarization of macrophages represents a pivotal immune response process within the host innate immune system, playing a fundamental role in maintenance of lung tissue homeostasis and modulation of inflammatory responses to pathogenic microorganism. Macrophages are generally believed to polarize into the M1 state after engulfing pathogens. In this state, they present viral peptides to T cells through the major histocompatibility complex (MHC), which in turn produce IFN-γ and other molecules to recruit effector immune cells and induce an inflammatory response to control viral replication [6,7]. Subsequently, an M2-like polarization is established to prevent excessive inflammation within the damaged tissue. For instance, after hosts were infected with highly pathogenic H5N1, the changes of human blood monocyte-derived macrophages subtype population were significant over time during the course of infection [8].

CD8^+^ and CD4^+^ T cells play pivotal roles in the human adaptive immune response against influenza infection, and persistent alterations in their function and subset composition following infection have significant implications for long-term prognosis. CD8^+^ or CD4^+^ T cells recognize viral antigens presented on MHC-I or MHC-II through their TCR with the assistance of antigen-presenting cells. However, the mechanisms underlying T cell activation in the pulmonary infection microenvironment remain elusive.

Antigen presentation is the pivotal process by which the immune system identifies and differentiates between endogenous and exogenous antigens. There are two primary types of antigen presentation. The conventional antigen presentation via MHC-I presents endogenous antigens primarily, such as viral or tumour antigens expressed within cells [9]. These antigens are degraded by the proteasomes into short peptides (8-11 amino acids) typically and transported to the endoplasmic reticulum (ER) by the transporter associated with antigen processing (TAP) subsequently [10]. The antigen peptide-MHC-I complex formed in the ER is then transported to the cell surface to activate the CD8^+^ T cell response. In contrast, exogenous antigens, such as protein antigens from inactivated vaccines, mainly presented through mechanisms mediated by MHC-II mainly. Endosomes or phagosomes, formed after the internalization of exogenous antigens by APCs, fuse with acidic MHC-II compartments (MIIC) in the cytoplasm, which are rich in various enzymes. These antigens are then degraded into short peptides (13–18 amino acids) [11, 12]. The peptide-MHC-II complex is transported to the cell surface, initiating the immune response of CD4^+^ T cells. For the antigen presentation via MHC-I, exogenous antigens from degrading bacteria or parasites can also be recognized by TCR of CD8^+^ T cells. MHC-I molecules present exogenous antigens through two main pathways: the TAP-dependent cytoplasmic pathway and the TAP-independent vesicle pathway [13, 14]. The study of cross-presentation has deepened the understanding of the link between the adaptive immunity of T cells and innate immunity.

Alveolar macrophages (AMs) are the predominant immune cells in the lungs and have been implicated in antigen presentation during fungal (*Cryptococcus neoformans*) and bacterial (*Mycobacterium tuberculosis*) infections in humans [15]. However, there is still a lack of supportive evidence for AMs-mediated antigen presentation in mice [16]. The conventional perspective suggests that AMs have poor capacity as viral antigen-presenting cells and lack the ability to migrate to lymph nodes, thereby limiting their involvement in priming naive T cells towards effector T cell differentiation [17]. Similarly, macrophages are often viewed as having a limited effect in antigen presentation and primarily maintaining homeostasis in the local pulmonary environment [18]. Although AMs are generally considered weak antigen-presenting cells, they can influence memory CD8^+^ T cells within the lungs by producing type I interferons (IFN) directly, even without the stimulation of cognate antigens [19]. Furthermore, it has been suggested that AMs impede the antigen-presenting function of lung dendritic cells (DCs) directly [20]. Given the intricate heterogeneity of macrophages, it is imperative to gain a deeper comprehension of the molecular mechanisms underlying antigen presentation during the polarization process.

Revolutions in the comprehension of cellular processes in disease are being brought about by single-cell RNA sequencing (scRNA-seq), which has greatly improved our ability to measure cell states at high resolution and scale. However, conventional scRNA-seq only captures static snapshots of gene expression at the experimental endpoint, fundamentally ignoring the temporal dimension. In mammalian cells, RNA levels are in a dynamic balance between synthesis and degradation. Therefore, conventional scRNA-seq methodologies that rely on total cellular RNA encounter limitations, such as inadequate temporal resolution to capture rapid transcriptional changes occurring within several hours and the inability to discern alterations in transcription, RNA processing and decay [21, 22]. Time-resolved scRNA-seq introduces a number of methods, such as Zman-seq. This single-cell technique tracks circulating immune cells in tissues for days by introducing a time stamp, recording transcriptome dynamics, and enabling empirical measurements of differentiation trajectories [23]. Time-resolved scRNA-seq using metabolic RNA labelling is also an excellent method for studying viral infections. Combining scRNA-seq with metabolic markers of new mRNA molecules allows for a more direct measurement for capturing RNA dynamics while overcoming common challenges accurately, such as the extended half-life of mammalian mRNA [24] and cellular heterogeneity that extends beyond transcriptional changes induced by viral infection stimulation [25], glucocorticoid receptor activation and T cell responses [21].

In this study, our objective was to investigate the dynamic transcriptional changes in different polarized macrophage states upon influenza A virus infection using single-cell thiol-(SH)-linked alkylation sequencing with RNA metabolic labelling of 4-thiouridine (s^4^U). Furthermore, we present novel evidence for the quantitative changes in conventional and cross-antigen presentation among distinct polarized macrophage states. These findings provide valuable insights into the pathways of antigen presentation in macrophages exhibiting diverse polarization states, thereby contributing to a comprehensive understanding of the antiviral response process in macrophages.

## Results

### The infectivity of influenza A virus in polarized macrophages exhibits significant heterogeneity, with M2 polarization demonstrating higher susceptibility

**Figure 1.**
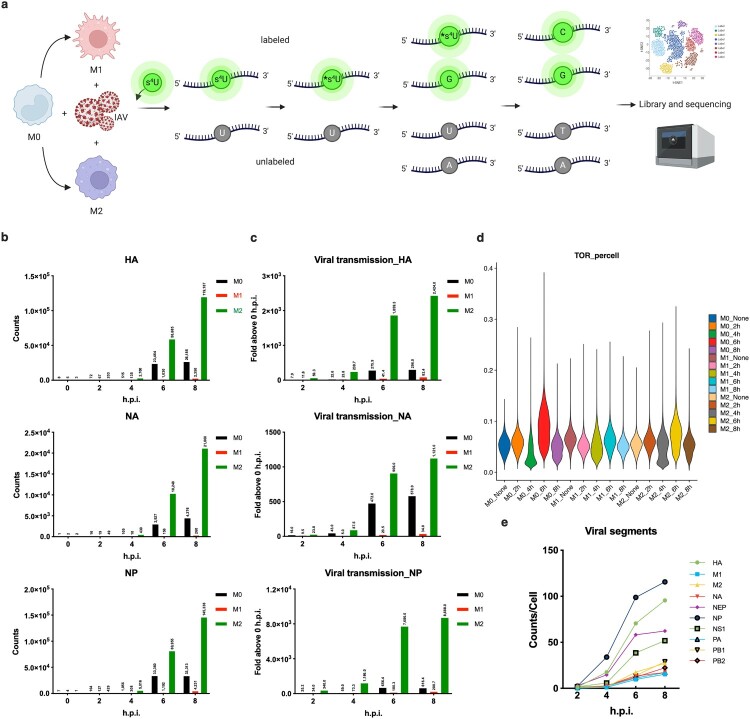
Workflow diagram of time-resolved scRNA-seq with s^4^U and significant heterogeneity of infection among different polarizations of BMDMs. (a) Process diagram of single-cell thiol-(SH)-linked alkylation of RNA for metabolic labelling sequencing (scSLAM-seq) method. (b) The levels of PR8 gene reads (HA, NA and NP) among M0, M1 and M2-BMDMs post IAV infection. M0, black; M1, red; M2, green. (c) Rates of viral infection spread changes (HA, NA and NP) in M0, M1 and M2-BMDMs post IAV infection. M0, black; M1, red; M2, green. (d) Conversion rates of new RNA in M0, M1 and M2-BMDMs post IAV infection. (e) PR8 viral transcriptional activity in *Arg1*_macrophages of M2-polarized BMDMs.

Dissecting the refined subsets of macrophages at a single-cell level poses significant challenges, yet it holds great potential for enhancing our understanding of antiviral immunopathogenesis. The two primary states of macrophage polarization, M1 and M2, play crucial roles in antiviral immunity, respectively. We hypothesized that heterogeneity exists in influenza A virus infection among macrophages in different polarization states. To test this hypothesis, macrophages were derived from the bone marrow of healthy adult mice and induced to differentiate into M1 or M2 phenotypes, while those not induced were considered to be in the M0 state. Subsequently, macrophages from the three distinct states were exposed to A/PR/8/34 (PR8) at a multiplicity of infection (MOI) of 1 for 1 h. After thorough washing with PBS, cells were collected immediately represented the 0-hour post-infection (h.p.i.) group, while the remaining cells were cultured with RNA metabolic markers in s^4^U-supplemented culture medium. Alongside a control group without viral inoculation, samples at different time points were processed into single-cell suspensions for dynamic transcriptome sequencing using a microfluidic chip platform. The thiol-reactive compound iodoacetamide (IAA) undergoes a nucleophilic substitution (S*_N_*2) chemical reaction with s^4^U, resulting in the attachment of a carboxyamidomethyl group to the thiol group of s^4^U [26]. Newly synthesized RNA will be labelled with the cytosine, while the pre-existing unlabelled RNA will retain thymine. The conversion rate of reverse transcription-dependent thymine to cytosine (T > C) was used to discern the dynamics of new and old RNA at a single nucleotide resolution (Figure 1a).

The results demonstrated a high degree of heterogeneity in the viral infection of BMDMs with different polarization states. Subsequent statistical analysis revealed significantly higher transcription readings for ten viral segments (e.g. HA, NA and NP) in the M2 state, lower levels of transcription readings in the M0 state, and extremely low transcription readings in the M1 state (Figure 1b and Fig.S1). Furthermore, we assessed the virus transmission rate by determining the number of infected cells capable of transcribing viral genes. Our findings indicated that PR8 strain exhibited stronger infectivity in the M2 state compared to both the M0 and M1 states (Figure 1c and Fig.S2). Additionally, the transcription levels of all viral genes increased over time within the M2 state specifically, suggesting an inability of this polarization state to restrict the infection of PR8. Conversely, it was observed that M1 polarization could limit viral replication more effectively.

To further elucidate the heterogeneity of influenza A virus infection in macrophages, we investigated the transcriptional dynamics of genes in distinct cellular states. Remarkably, our findings demonstrate that 6 h post-infection represents a critical time point for virus-host interactions, as evidenced by the peak period of new RNA synthesis observed in M0, M1 and M2 host cells (Figure 1d). Notably, this temporal pattern aligns with an upregulation of viral gene transcription, particularly in the most susceptible M2 state. Furthermore, we identified differential transcription rates among individual genome segments of PR8 strain, which can be categorized into two groups broadly: HA, NP, NS1 and NEP (NS2), which exhibit a rapid increase followed by a gradual decline starting at 4 h post-infection. By contrast, other viral genes such as PA, PB1 and NA display a peak at 6 h followed by subsequent decrease (Figure 1e). Collectively, these results suggest that among infected *Arg1*_macrophages, PR8 hijacks host machinery to synthesize HA, NP and NS while concurrently splicing into NEP (NS2) preferentially, and other viral genes are transcriptionally synthesized at a lower speed.

### The state of *Arg1*_macrophage represents the exclusive cluster which establishes a permissive environment for the replication of PR8

**Figure 2.**
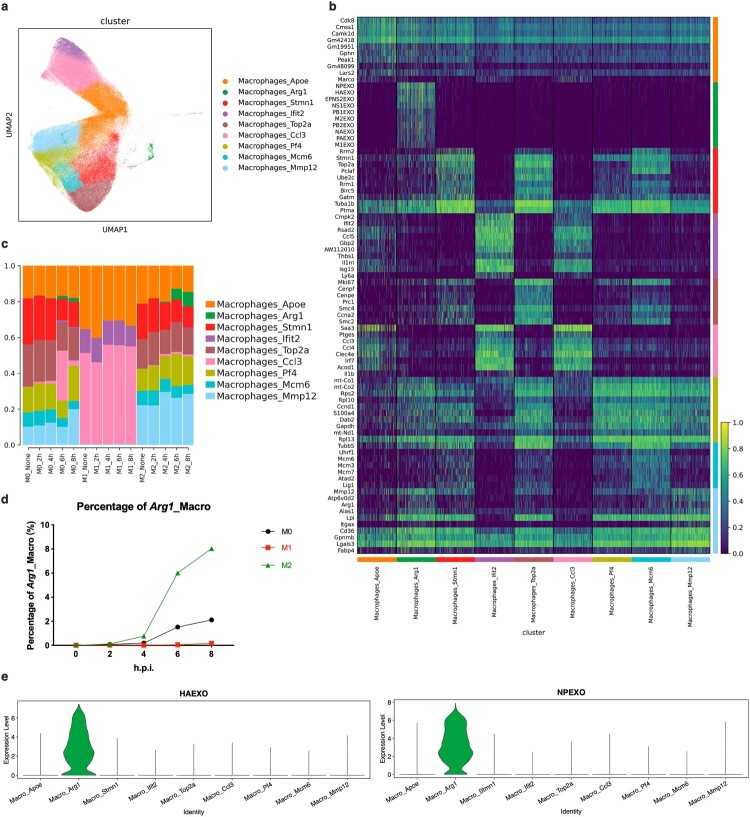
*Arg1*_macrophage cluster is the unique cluster of BMDMs which establishes a permissive environment for the replication of PR8 strain. (a) State subsets division for *Apoe*, *Arg1*, *Stmn1*, *Ifit2 Top2a*, *Ccl3*, *Pf4*, *Mcm6* and *Mmp12*_macrophages. (b) Heat-map of gene expression differences among every polarized states of BMDMs. (c) Dynamic proportion changes of every subset in M0, M1 and M2-polarized BMDMs post IAV infection. (d) Dynamic percentage of *Arg1*_macrophages in M0, M1 and M2 post infection. (e) Expression levels of viral genes HA and NP among different macrophage state clusters.

In order to further investigate the intrinsic factors contributing to the heterogeneity of M0, M1 and M2 post infection, we employed the uniform manifold approximation and projection (UMAP) algorithm for a more comprehensive classification of macrophage subpopulation states. Our study revealed that the subdivision into nine distinct subpopulations clusters encompassed all three polarized states of infected BMDMs effectively. These subpopulations were denoted as *Apoe*_macrophages, *Arg1*_macrophages, *Stmn1*_macrophages, *Ifit2*_macrophages, *Top2a*_macrophages, *Ccl3*_macrophages, *Pf4*_macrophages, *Mcm6*_macrophages and *Mmp12*_macrophages (Figure 2a). The division of the nine clusters of BMDMs is detailed in Figure 2(b).

*Apoe* encodes apolipoprotein E, and study has shown that lower inflammation in the lung leads to the development of ApoE-dependent monocyte-derived AMs (ApoE^+^CD11b^+^), as a key factor of tissue resilience [27]. *Arg1* encodes arginase 1, which hydrolyzes L-arginine to urea and ornithine, participates in lung tissue damage repair, and is an important marker of M2-like polarization [28]. *Stmn1* encodes stathmin 1, which has been identified as strongly associated with the cell cycle in macrophages from the lungs of mice with in situ breast tumours [29]. *Ifit2* encodes IFN-induced protein with tetratricopeptide repeats 2, it can be used as a key effector of LPS-induced secretion of TNF-α and endotoxic shock in BMDMs [30]. *Top2a* encodes type IIA DNA topoisomerase, it catalyzes the breaking and joining of dsDNA to change its topology [31]. CCL3, also known as MIP-1α, is encoded by Ccl3 and is known to regulate chemotaxis, polarization and function of M1-like macrophages, for example by exacerbating inflammatory bowel injury [32]. Platelet factor 4 is encoded by *Pf4*. It has been reported that *Pf4*^+^ macrophages in the late stage of IAV infection may be precursors of alveolar macrophages (AMs) [33]. The proteins encoded by *Mcm6* and *Mcm12* are highly conserved mini-chromosome maintenance (MCM) proteins that are essential for the initiation of replication of the eukaryotic genome [34].

Notably, state subset *Arg1*_macrophages facilitated influenza A virus replication exclusively. Dynamic sequencing analysis showed that *Apoe*_macrophages represented the subgroup state shared by M0, M1 and M2 in the absence of infection (Figure 2c). The predominant components of M0 macrophages were *Apoe*, *Stmn1*, *Top2a*, *Pf4*, *Mcm6* and *Mmp12*_ macrophages. Upon influenza virus infection, the *Ccl3* subgroup gradually emerged and reached its peak activity at 6 h.p.i., followed by a rapid decline. The composition of M1 macrophages remained largely unchanged after infection with *Apoe*, *Ifit2* and *Ccl3*_ macrophages being the major constituents. In contrast to M0 and M1 states, although the proportion of *Ccl3* state subsets did not significantly increase in infected M2 macrophages; and a corresponding increase in *Arg1*_cluster was observed instead (Figure 2c). Notably from the gene expression heat-map analysis, genes related to influenza A virus replication exhibited high transcriptional activities primarily within the *Arg1*_macrophages cluster (Figure 2b).

Therefore, the observed heightened susceptibility of M2 macrophages primarily stems from their inherent ability to readily induce *Arg1*_cluster following infection, while inducing *Arg1*_cluster in M1 polarization is considerably challenging, thus demonstrating their capacity to restrict viral replication (Figure 2d). The capability of M0 macrophages to induce *Arg1*_cluster upon exposure to the influenza A virus lies between that of M1 and M2. The replication permittivity of *Arg1*_cluster was further confirmed in the violin plot, and the all eight segmented genes of PR8, including HA and NP, were predominantly enriched within the *Arg1*_macrophages (Figure 2e and Fig.S3). Thus, *Arg1*_macrophages represent a state susceptible for viral infection pleasantly.

We conducted a detailed analysis of Arg1_macrophages, the cluster in which the PR8 strain can replicate, in M2-polarized macrophages. Firstly, we observed a significant down-regulation of ribosome-associated genes over time after infection, particularly evident after 6 h.p.i. (Fig. S5a). The ribosome, a complex molecular machine crucial for protein synthesis, consists of various ribosomal proteins and ribosomal RNA, playing a central role in gene expression. The pronounced downregulation of ribosome-related genes suggests that the virus interferes with normal ribosome synthesis in M2-polarized macrophages post-infection. Secondly, we categorized the genes in *Arg1*_cluster that change after infection into nine groups based on their temporal dynamics. Specifically, we examined the 7th group, characterized by peak gene expression at 6 h.p.i. (Fig. S5b). Subsequent analysis revealed a set of genes deeply involved in mitochondrial electron transport, including *Cox1*, *Cox2*, *Cox3*, *Nad1*, *Nad2*, *Nad4*, and *ATPase-6*. Mitochondria are crucial energy-generating organelles, and the impaired function of the mitochondrial respiratory chain in the *Arg1*_macrophages cluster suggests a substantial energy contribution to viral replication.

### Antigen cross-presentation abilities to CD8^+^ T cells exhibited heterogeneity among BMDMs with distinct polarized states

It has traditionally been believed that cross-presentation is primarily accomplished by dendritic cells (DCs), meaning DCs can effectively mediate the presentation of pathogens from non-infected DCs, while macrophages possess weaker abilities in this regard. In this study, we demonstrated that influenza A virus exhibit distinct life cycles within different polarized states of host macrophages with M1 polarization exhibits a robust capacity to restrict influenza virus replication. This suggests that M1 polarization may possess a strong capability to phagocytize pathogens as exogenous antigens and subsequently cross-present them to CD8^+^ T cells. Therefore, we investigated the cross-presentation of CD8+ T cells by macrophages with diverse polarization states.

**Figure 3.**
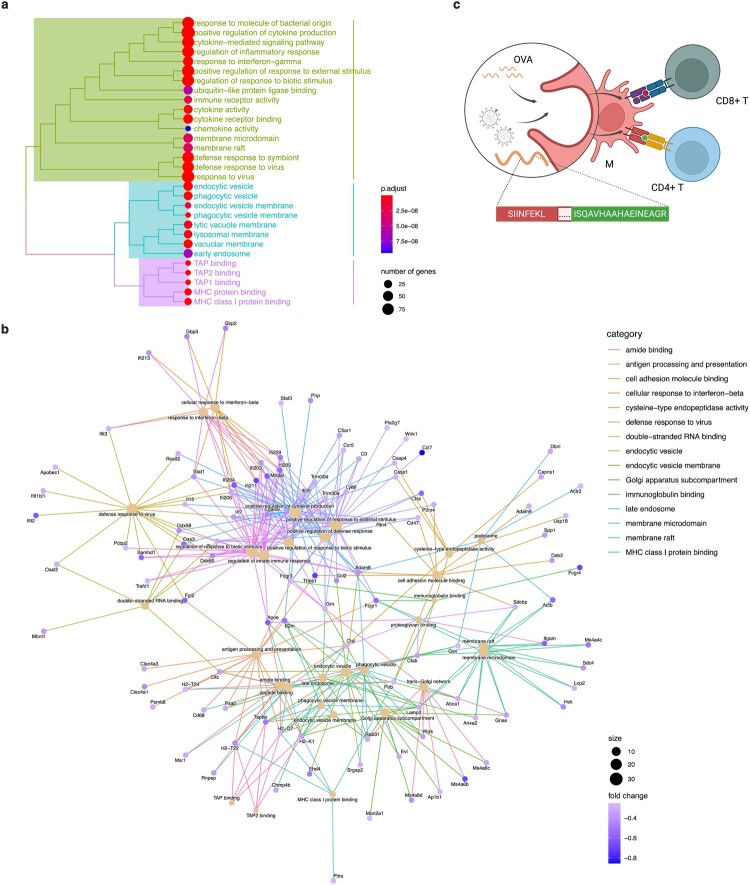
Macrophages with distinct polarized states exhibited heterogeneity in their antigen cross-presentation abilities to CD8^+^ T cells. (a) GO enrichment change analysis of molecular function (peak green), cellular component (blue) and biological process (purple) in *Ccl3*_macrophages. TAP binding and MHC-I proteins related genes were found to be up-regulated significantly. (b) Network communication analysis of down-regulated genes in *Arg1*_macrophage cluster. (c) Diagram of antigen presentation experiments through macrophages.

**Figure 4.**
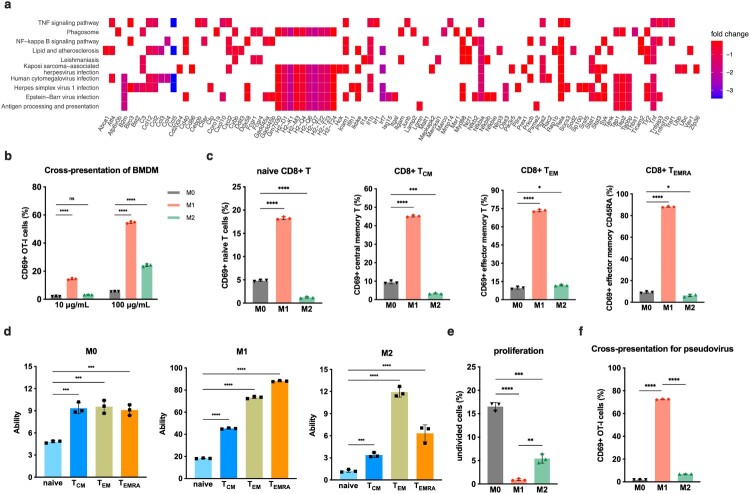
(a) Heatmap of down-regulated genes changes in *Arg1*_ macrophages. (b) Cross-presentation of M0, M1 and M2-BMDMs with pulsed DQ-OVA. (c) Cross-presentation to naive CD8^+^ T cells, CD8^+^ T_CM_, CD8^+^ T_EM_ and CD8^+^ T_EMRA_. (d) M0, M1 and M2 respective cross-present to naive CD8^+^ T cells, CD8^+^ T_CM_, CD8^+^ T_EM_ and CD8^+^ T_EMRA_. (e) Proliferation of CD8^+^ T cells with ovalbumin incubation. (f) Antigen cross-presentation of M0, M1 and M2 in alveolar macrophages (AMs) from human ACE2 gene knock-in mouse with infection of SARS-CoV-2 pseudovirus at MOI = 1. The statistical significance was analysed by ANOVA test: *p<0.05, **p<0.01, ***p<0.001 and ****p<0.0001.

We first enhanced the data of dynamic transcriptome sequencing. As a constituent of the extensive and distinctive M1 subgroup, *Ccl3*_ macrophages exhibited up-regulation of various signalling pathways that mutually activate CD8^+^ T cells, including MHC-I binding proteins and transporter associated with antigen processing (TAP), in comparison to other substates (Figure 3a). Conversely, as the sole influenza A virus capable of replicating *Arg1*_ macrophages, it down-regulated a range of antigen processing and presentation genes such as *Tap1*, *Tap2* and *Tapbp*, along with MHC-I related genes *H2-D1*, *H2-K1*, *H2-Q6* and *H2-Q7* significantly (Figure 4a). Additionally observed was an enrichment in endosomes and other crucial organelles which influencing viral antigen-presentation through network communication analysis (Figure 3b).

Then, we assessed the abilities of BMDMs for cross-presentation in distinct states through antigen presentation experiments (Figure 3c). DQ-OVA is a self-quenching OVA conjugate that, when hydrolyzed in cells, fluoresces BODIPY and is insensitive to pH changes [35]. Our findings revealed that M1 polarization exhibited a robust antigen cross-presentation ability following pulsed stimulation with DQ-OVA, while the M0 resting state displayed the weakest capability. Moreover, under low antigen concentrations, there was no significant disparity in the cross-presenting ability between M2 and M0. However, as antigen concentration increased, M2 polarization demonstrated a substantial enhancement in cross-presentation on total CD8^+^ T cells (Figure 4b). In accordance with the linear differentiation theory, we also investigated how macrophages in different states influenced CD8^+^ T cell activation at various stages of differentiation. Notably, our studies consistently demonstrated that M1 possessed the strongest cross-presentation ability across all stages of CD8^+^ T cell differentiation. Interestingly, for naive CD8^+^ T cells, central memory CD8^+^ T cells (CD8^+^ T_CM_) and effector memory re-expressing CD45RA^+^ CD8^+^ T cells (CD8^+^ T_EMRA_), the order of cross-presentation ability was observed as follows: M1 > M0 > M2. However, M2 exhibited slightly higher potency compared to M0 for effector memory CD8^+^ T cells (CD8^+^ T_EM_) specifically (Figure 4c).

Simultaneously, data analysis also revealed that M1 polarized macrophages exhibit enhanced activation intensity towards the CD8^+^ T cell subpopulation in the order of T_EMRA_ > T_EM_ > T_CM_ > naive, indicating an augmented cross-presentation ability with increasing differentiation of CD8^+^ T cells. Conversely, for M2 polarization, CD8^+^ T_EM_ demonstrated the highest potency followed by CD8^+^ T_EMRA_ and CD8^+^ T_CM_, while naive exhibited the weakest capability. Notably, there were no significant differences observed in resting state M0 among memory T cells (CD8^+^ T_CM_, T_EM_ and T_EMRA_), with all exhibiting superior presentation compared to naive CD8^+^ T cells (Figure 4d). Overall, macrophages display weaker presentation abilities than memory T cells. What is noticeable is that even the weakest cross-presentations by M1 surpasses those of both M0 and M2 at their strongest levels. Furthermore, CD8^+^ T cell proliferation supported these findings as incubation with ovalbumin resulted in a lower proportion of undivided cells for M1 compared to higher proportions for M0 and intermediate proportions for M2 (Figure 4e). Similarly, we generated a pseudovirus system of SARS-CoV-2 using vesicular stomatitis virus (VSV) as a vector with the modification of specific antigenic peptides on spike glycoprotein. Upon infected the different states of murine alveolar macrophages (AMs) at the multiplicity of infection of 1 (MOI = 1), it was observed that cross-presentation by M1 to SARS-CoV-2 pseudovirus was greater than that by both M2 and M0 significantly (Figure 4f). To sum up, our results indicate that highly differentiated memory CD8^+^ T cells are preferentially presented by macrophages due to their efficient cross-presentation ability.

### M1 state macrophages demonstrated the most efficient conventional antigen presentation to CD4^+^ T cells

After the completion of MHC-II molecular loading with exogenous antigen peptides, secretory vesicles containing pMHC-II are formed. Subsequently, through fusion events between these vesicles and the cell membranes, pMHC-II molecules are expressed on the cell surface for recognition by CD4^+^ T cells. This represents the classical pathway for processing and presenting exogenous antigens. Furthermore, we investigated the heterogeneity of conventional antigen presentation among distinct polarized macrophage towards CD4^+^ T cell subsets.

The results demonstrated that, in comparison to M0 and M2, M1 exhibited the highest capacity for direct presentation of exogenous antigens to CD4^+^ T cells (Figure 4a). Furthermore, within naïve CD4^+^ T, CD4^+^ Treg, and all memory CD4^+^ T cell subsets, the direct presentation ability of M1 was superior to that of M0 and M2 significantly (Figure 4b). However, it is also noteworthy that the direct presentation strength of M1 towards CD4^+^ T cells is inferior to its cross-presentation capability towards CD8^+^ T cells. Additionally, it is intriguing to observe that different subpopulations of CD4^+^ T cells exhibit varying conventional presentation abilities by M1. The data revealed a high efficacy of M1 in presenting antigens to CD4^+^ T_EM_ and Treg subsets directly, followed by CD4^+^ T_EMRA_ and CD4^+^ T_CM_ subsets. In contrast, the direct presentation capacity towards naive CD4^+^ T cells was remarkably weak. Similar to M1, both M0 and M2 presented a significantly higher proportion of CD4^+^ Treg compared to various memory CD4^+^ T cells; however, neither of them was able to effectively activate naive CD4^+^ T cells directly (Figure 4c). In conclusion, macrophages polarized under all states can robustly activate Tregs while only M1 exhibits strong antigen presentation capabilities towards the CD4^+^ T_EM_ subset as well. Despite this, M0, M2, or even M1 are unable to directly present foreign antigens to the initial CD4^+^ T cells. The infection of murine alveolar macrophages with modified SARS-CoV-2 pseudovirus also confirmed that M1 polarization exhibited the highest presentation to CD4^+^ T cells, albeit still lower than its CD8^+^ T cell presentation level (Figure 4d).

**Figure 5.**
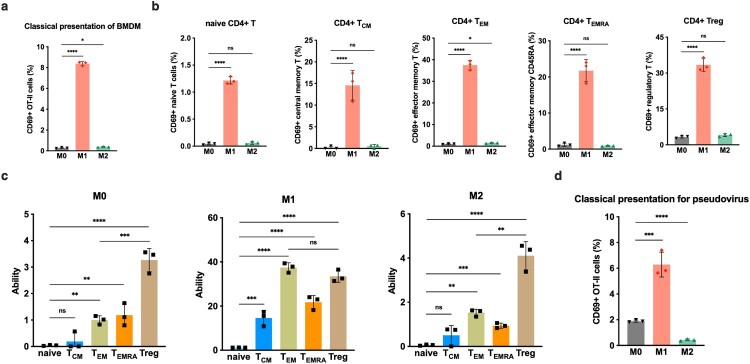
M1-polarized macrophages demonstrate the most efficient conventional antigen presentation to CD4^+^ T cells. (a) Conventional antigen presentation to total CD4^+^ T cells of M0, M1 and M2-BMDMs with pulsed DQ-OVA. (b) Antigen classical presentation to naive CD4^+^ T cells, CD4^+^ T_CM_, CD4^+^ T_EM_, CD4^+^ T_EMRA_ and CD4^+^ Treg of M0, M1 and M2-BMDMs with pulsed DQ-OVA. (c) M0, M1 and M2 respective classical presentation to naive CD4^+^ T cells, CD4^+^ T_CM_, CD4^+^ T_EM_, CD4^+^ T_EMRA_ and CD4^+^ Treg. (d) Conventional antigen presentation of M0, M1 and M2 in alveolar macrophages from human ACE2 gene knock-in mice with infection of SARS-CoV-2 pseudovirus at MOI = 1. The statistical significances were analysed by ANOVA test: *p<0.05, **p<0.01, ***p<0.001 and ****p<0.0001.

We further investigated the regulation of lysosomal protein activity in mouse macrophages. Comparative analysis revealed that M2 and M0 (BMDMs) exhibited elevated expression of lysosomal genes and enhanced degradation of DQ-OVA, as compared to M1 BMDM. Similarly, increased degradation of DQ-OVA was observed in M2-polarized alveolar macrophages (Figure 5a). These findings provide evidence for heightened lysosomal enzyme activity in M2-polarized macrophages. Moreover, phagocytosis represents a major function of macrophages. Our engulfment experiments confirmed no significant differences in phagocytic capacity among the three states of both BMDM and alveolar macrophages (Figure 5b), which was similar to the human peripheral blood mononuclear cell-derived macrophages previous report [36]. This also suggested that disparities in antigen conventional or cross-presentation ability were not attributable to variations in antigen phagocytosis of different macrophages states.

**Figure 6.**
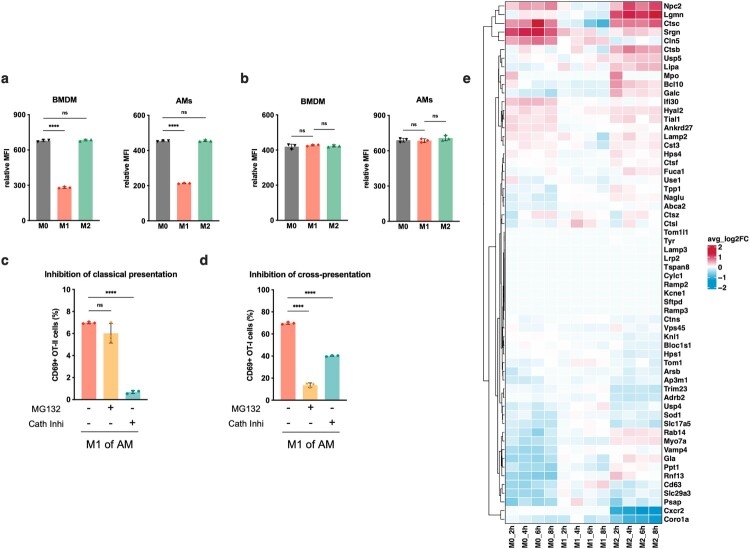
Degradation and phagocytic assays and lysosomal genes in M0, M1 and M2-BMDMs post IAV infection. (a) DQ-OVA degradation assays of M0, M1 and M2 in BMDMs and murine alveolar macrophages. Relative MFI, mean fluorescence intensity fold above the control group. (b) Phagocytic activity of M0, M1 and M2 in murine alveolar macrophages and BMDMs following incubation with fluorescent microbeads for 1 h. Relative MFI, mean fluorescence intensity fold above the control group. (c) Effect of inhibitors with MG132 or cathepsin inhibitor I on conventional presentation to CD4^+^ T cells in M1 of murine alveolar macrophages. (d) Effect of inhibitors with MG132 or cathepsin inhibitor I on cross-presentation to CD8^+^ T cells in M1 of murine alveolar macrophages. (e) Heatmap of genes expression difference among M0, M1 and M2-polarized BMDMs changes over time. The statistical significances were analysed by ANOVA test: *p<0.05, **p<0.01, ***p<0.001 and ****p<0.0001.

It is widely accepted that dendritic cells employ two distinct cellular pathways, namely the cytoplasmic pathway and vacuole pathway, for antigen cross-presentation. However, there is a lack of available data on the cross-presentation mechanism of alveolar macrophages in mice. Pretreatment of M1 alveolar macrophages with the proteasome inhibitor MG132 had minimal impact on CD4^+^ T cell presentation but did not completely inhibit activation of epitope-specific CD8^+^ T lymphocytes, indicating a partial reliance on proteasome processing in M1 cross-presentation. Conversely, inhibition of cathepsins using a broad-spectrum inhibitor significantly reduced both conventional CD4^+^ T cell presentation and cross-presentation, suggesting the essential role of the vacuole pathway in M1 presentation, particularly for cross-presentation (Figure 5c and d). Collectively, these findings suggest that M1-polarized alveolar macrophages primarily utilize the vacuolar pathway to present antigens to CD8^+^ T cells while also potentially utilizing cytosolic pathway Figure 6.

## Discussion

Macrophages possess remarkable plasticity and exhibit distinct characteristics and functions within intricate environments. Classically activated macrophages (M1) and alternatively activated macrophages (M2) represent the two predominant polarization phenotypes of macrophages. The cytokines and chemokines profiles as well as their corresponding functions differ significantly between M1 and M2. Due to the distinct polarization states, the diverse and complementary functions of this phenomenon sustain the normal functioning of the systemic immune system, regulate tissue homeostasis and inflammation, thereby effectively safeguarding overall bodily well-being. However, the systematic investigation of cross-presentation of exogenous antigens by primary M0, M1 and M2 macrophages in comparison to conventional presentation to various T cell subpopulations remains unexplored.

In this study, we employed a novel dynamic single-cell sequencing method to demonstrate the ineffectual replication capacity of influenza A virus within each state subgroup of M1 type. This observation suggested that influenza A virus is processed as exogenous antigens upon infecting M1 phenotype predominantly. Consequently, our investigation focused on discerning the disparities in exogenous antigen presentation between M1 and M0 or M2.

Due to the infective bias of many viruses and bacteria against different cells, as well as their inability to infect antigen-presenting cells (APCs), host CD8^+^ T cells cannot effectively monitor all pathogens solely relying on conventional antigen-presenting pathways. The antigen cross-presentation pathway enables CD8^+^ T cells to participate in the elimination of viruses, bacteria, and tumour immunity even when they cannot infect APCs. It was widely acknowledged that DCs possess superior antigen cross-presenting ability among APCs, while macrophages exhibit relatively weaker capacity due to their enhanced lysosomal protein degradation capability [17, 37]. Similarly, it was regarded that alveolar macrophages exhibited a relatively poor level for antigen presentation [38].

However, based on our comprehensive sequencing analysis and rigorous experimental verification, it was observed that M1 BMDMs exhibited a remarkably robust capacity for cross-presenting exogenous antigen OVA compared to both M2 and M0 phenotypes. Notably, mouse alveolar macrophages in the M1 state also demonstrated highly efficient cross-presentation of the SARS-CoV-2 pseudovirus to pathogen-specific CD8^+^ T cells. These findings strongly suggest that macrophages in the M1 phenotype are proficient at initiating antiviral CD8^+^ T cell responses. It should be noted that the capacity of M1 macrophages to present to each subgroup of CD8^+^ T cells is not uniform. In advanced stages of memory differentiation, M1 BMDMs exhibit stronger cross-presentation levels for CD8^+^ T cells, while naive CD8^+^ T cells show lower levels. This suggests that M1 polarization has a significant advantage in initiating rapid memory and effector CD8^+^ T cell responses. Furthermore, the ability of M1 to cross-present naive CD8^+^ T cells surpass that of both M0 and M2 in presenting memory CD8^+^ T cells; thus, it cannot be overlooked.

In the conventional MHC-II presentation pathway, APCs internalize exogenous pathogen proteins through phagocytosis or endocytosis and subsequently engage in CD4^+^ T cell presentation, a specialized function performed by professional APCs [39]. We also focused on the direct presentation of exogenous antigens by macrophages in different states. Firstly, overall M1- polarized BMDMs still exhibit the strongest classical polarization for antigen presentation. However, the conventional presentation of exogenous antigens in M1 is considerably weaker compared to cross-presentation. This conclusion was further supported by experiments demonstrating that alveolar macrophages presenting the SARS-CoV-2 pseudovirus to total CD4^+^ T cells exhibited similar findings. Secondly, M1 macrophages demonstrated a robust ability to present antigens to Treg cells, aligning with observations made for both M0 and M2 macrophage subsets, indicating excellent Treg activation potential across all three states. Thirdly, regardless of whether it is the M1, M0, or M2 state, all three subsets displayed limited capacity for presenting exogenous antigens to naive CD4^+^ T cells. Additionally, the CD4^+^ T_EM_ presentation capability of M1 was comparable to that observed for CD4^+^ Treg; overall, conventional antigen presentation by differentiated stages of CD4^+^ T cell subsets was enhanced as differentiation progressed.

Cross-presentation involves the uptake of extracellular antigens, processing them into peptides, and presenting these peptides on MHC-I molecules to CD8^+^ T cells [40]. In terms of phagocytosis, no discernible difference was observed between BMDMs and alveolar macrophages in the three states examined. However, only M1 macrophages exhibited a relatively higher degradation capacity towards DQ-OVA, implying that excessive lysosomal protein degradation may not favour antigen presentation in macrophages. Whereas BMDMs (which grown in M-CSF can be considered M0) exhibited elevated levels of lysosomal proteases and efficiently degraded internalized proteins, while BMDCs displayed a limited presence of proteases, resulting in thus retained antigen for extended periods for a powerful antigen presentation [37]. Cathepsin inhibitor I nor MG132 which were used for inhibiting vacuolar and cytosolic cross-presentation pathway, respectively [41]. In this study, we found that neither cathepsin inhibitor I nor MG132 completely abrogated the cross-sensitization of alveolar macrophages; however, cathepsin inhibitor I significantly inhibited CD4^+^ T cell presentation while MG132 did not, suggesting that cross-presentation by alveolar macrophages involves both cytoplasmic and vacuolar pathways whereas direct presentation primarily relies on the vacuolar pathway. What’s more, constrained lysosomal proteolysis also facilitated antigen presentation of M1.

In addition, we also proposed that timely modulation of M1/M2 macrophage polarization is crucial for antigen-specific CD8^+^ T cell responses during the course of the influenza disease. In the early stages of viral invasion, an inflammatory response mediated by M1 macrophages aids in pathogen clearance, followed by a shift towards M2 dominance to facilitate tissue repair [42]. And persistent predominance of M1 polarization which supported the paradigm of a persistent proinflammatory state can lead to immunopathological lung damage [43]. CD8^+^ T_EM_ and CD8^+^ T_EMRA_ are not only enriched in the lungs, but also play an important role in anti-respiratory virus immunity. A study that tracked T cell status in 342 healthy adults during the 2009 UK influenza pandemic found the strongest inverse association between IFN-γ^+^IL-2^-^CD8^+^ T cell frequency and disease symptom score (*r* = −0.6, *P* = 0.004), and almost all of these protective CD8^+^ T cells exhibited CD8^+^ T_EM_ and CD8^+^ T_EMRA_ phenotypes [44]. Long-term studies of the CoronaVac vaccine have shown that CoronaVac was still very protective 12 months after the second boost, it was because it induced persistent SARS-CoV-2-specific CD8^+^ T_EM_ and CD8^+^ T_EFF_ (T_EMRA_) to still produce IFN-γ, IL-2 and GzmB against viral RBD [45]. Treatment studies in patients with severe H7N9 have shown that their recovery is highly correlated with a CD8^+^ T-cell-led immune response, especially with the recovery of the virus-specific CD8^+^ T_EMRA_ that coincides with viral clearance during recovery [46]. When CD8^+^ T_EM_ or CD8^+^ T_EMRA_ is abnormal, it is not conducive to the prognosis of the infected host. Clinical studies have found that up to 80% of CD8^+^ T_EM_ in the blood of severe COVID-19 patients is in a depletion cycle near death, and it was easy to go to apoptosis [47]. In addition, the proportion and count of CD8^+^ T_EM_ in the peripheral blood of patients with long-term positive COVID-19 (mean negative nucleic acid transition time of up to 66.2 days) were significantly reduced, and the response of IFN-γ^+^CD8^+^ T cells specific to the viral nucleocapsid was very weak. This indicated that CD8^+^ T_EM_ response was severely inhibited in patients [48]. And during the acute phase of influenza infection, enhanced cross-presentation of CD8^+^ T_EM_ and CD8^+^ T_EMRA_ by highly inflammatory infiltrating M1 macrophages in lung may lead to excessive cross-priming and promote T-cell apoptosis due to the strong presentation.

The novelty of this study lies in its systematic report on the impact of macrophage status changes on antigen presentation to CD4^+^ T and CD8^+^ T cell subpopulations, with a particular emphasis on the prominent role of M1 polarization in cross-presentation, especially in primary alveolar macrophages. Our findings contribute to a deeper understanding of the significance of macrophage polarity remodelling in antiviral T cell immunity within the lung. Antigen-presenting cells play a pivotal role in the management of respiratory virus infections. Further investigation and comprehension of the intricate interactions between antigen-presenting cells and the immune system are imperative. A comprehensive examination of diverse macrophage states will facilitate a deeper understanding of the pathogenesis associated with disease caused by virus-specific T cell immune responses, thereby establishing essential theoretical foundations and potential drug targets for innovative clinical strategies aimed at prevention and treatment.

## Experimental methods

Cell extraction and cultures. MDCK cells and L929 cells were cultured in DMEM (Dulbecco’s modified Eagle’s medium) supplemented with high glucose and L-glutamine (Gibco®) in addition to 10% FBS (fetal bovine serum) and 1% penicillin/streptomycin in a 5% CO_2_ incubator at 37°C. The supernatant of L929 cells was collected and stored at −80°C. The cell lines used are routinely tested for mycoplasma and are maintained mycoplasma-free.

Bone marrow-derived macrophages (BMDMs) were isolated from femur and tibia bones of 8-week-old mice. Bone marrow was flush out with the help of 1 mL syringe, and filtrated with 70 μm cell strainer. Then, red blood cell lysis buffer (Invitrogen) was added and cleaved at room temperature for 5 min. After centrifugation at 1,500 rpm for 5 min, the cells were re-suspended and cultured in fresh 80% RPMI 1640 medium supplemented with high glucose and L-glutamine (Gibco®) in addition to 10% heat-inactivated FBS and β-mercaptoethanol, and 20% supernatant of L929 cells in 5% CO_2_ incubator at 37°C. On day 3, use the same formula for a full liquid change. On day 6, fresh stimulation medium was replaced with RPMI 1640 medium containing 10% HI-FBS, 100 ng/mL LPS (Sigma) and 50 ng/mL murine IFN-γ (PeproTech) for M1 polarization; 10 ng/mL murine IL-4 (PeproTech) and 10 ng/mL murine IL-13 (PeproTech) for M2 polarization. Then the phenotypes of M1 (CD86^+^CD206^-^) and M2 (CD86^-^CD206^+^) were determined by flow cytometry, respectively. Murine alveolar macrophages were isolated as predecessors’ research, and optimized 37°C PBS with 2 mM EDTA and 0.5% FBS for harvest [49]. Pre-warm fresh RPMI 1640 medium with 20 ng/mL murine GM-CSF (PeproTech) and 100 ng/mL LPS and 20 ng/mL IFN-γ for M1 polarized; while 20 ng/mL IL-4 and 10 ng/mL IL-13 for M2 polarized at least 24 h.

Immune cells such as CD8^+^ T cells and CD4^+^ T cells were purified from fresh splenic single-cell suspension from OT-I mice or OT-II mice with the help of corresponding EasySep™ magnetic beads separation kit (Stemcell®). Then, cell debris of T cells were removed with dead cell removal kit (Stemcell®) and were static cultured overnight in a commercial ImmunoCult™-XF T cell expansion medium (Stemcell®) in 5% CO_2_ incubator at 37°C.

Viruses, pseudovirus and *h*ACE2 mouse. Influenza A virus strain used in the study was PR8 strain and all laboratory procedures involving live viruses were performed under biosafety level 2 (BSL-2) conditions. The influenza A virus strain was propagated and tittered on MDCK cells with specialized serum-free medium for influenza virus isolation (Yocon Biology) and serum-free medium for MDCK cells culture (Yocon Biology). For live influenza A virus infection, M0, M1 and M2 BMDMs were incubated with the indicated viral strain at MOI of 1 for 1 h at 37°C followed by two washes with PBS.

We employed a recombinant vesicular stomatitis virus (VSV) system in place of the original G protein to generate pseudovirus. We design the special base sequences to extra encode peptide sequence SIINFEKL (H-2K^b^ of MHC-I) tag or ISQAVHAAHAEINEAGR (I-A^d^ of MHC-II) on the envelope plasmid (spike protein with tag) and expression plasmid (spike protein with tag) of the lentiviral vector, and they were packaged by Vector Builder without the ability of replication in host cells, and the sequence of spike glycoprotein of SARS-CoV-2 is based on the previous study [50]. The *h*ACE2 knock-in mice were purchased from Beijing Biaopu Technology company which were prepared by using the CRISPR/Cas9 system, with the donor vector containing the homology arm sequence and the cDNA of human ACE2, and the specific sgRNA sequence was 5’-GAAAGATGTCCAGCTCCTCCTGG-3’.

Time-resolved scRNA-seq. For labelling experiments, according to the protocol of the DynaSCOPE® Single Cell Dynamic RNA Library Kits (Singleron), thaw Labeling reagent (Singleron) at room temperature, prepare labelling culture medium, add labelling reagent to medium at 1:100 ratio (labelling reagent: medium) and mix well. Transfer the prepared single cells to the labelling culture medium and keep the cell culture away from light. After incubation, discard supernatant and resuspend cell pellet in an appropriate volume of PBS. And then, Single-cell suspensions (2×10^5^ cells/mL) with PBS (HyClone) were loaded onto microwell chip using the Singleron Matrix® Single Cell Processing System. Barcoding Beads are subsequently collected from the microwell chip, followed by reverse transcription of the mRNA captured by the Barcoding Beads and to obtain cDNA, and PCR amplification. The amplified cDNA is then fragmented and ligated with sequencing adapters. The scRNA-seq libraries were constructed according to the protocol of the GEXSCOPE® Single Cell RNA Library Kits (Singleron) [51]. Individual libraries were diluted to 4 nM, pooled, and sequenced on Illumina NovaSeq 6,000 system with 150 bp paired end reads.

The turn-over rates (TOR) were calculated as proportion of newly synthesized transcripts in whole transcripts based on the counts of unique molecular identifier (UMI) in each gene or cell. The result was projected to the UMAP and *t*-SNE plots from Seurat clustering analysis for visualization consistency. Turn-over rates were also shown by Violin plots with statistical test. In addition, the high TOR genes were filtered to explore genes which had active states of transcription. Based on the newly synthesized transcripts, the high TOR genes were defined as not only gene turn-over rates > 0.1 but also expressing in more than 1% sample cells.

To identify the viral RNA in single cells, we aligned raw scRNA-seq reads using kallisto/bustools (KB) against a customized reference genome, in which the genome of PR8 strain: *HA*, NC_002017.1, FLUAVs4gp1; *NA*, NC_002018.1, FLUAVs6gp1; *PA*, NC_002022.1, FLUAVs3gp1; *PB1*, NC_002021.1, FLUAVs2gp1; *PB2*, NC_002023.1, FLUAVs1gp1; *NP*, NC_002019.1, FLUAVs5gp1; *M1*, NC_002016.1, FLUAVs7gp1; *M2*, NC_002016.1, FLUAVs7gp2; *NS1*, NC_002020.1, FLUAVs8gp2; *NEP (NS2)*, NC_002020.1, FLUAVs8gp1 (name, accession, aliases) from NCBI Reference Sequence Database was added as an additional chromosome to the mice reference genome. A single cell with either viral RNA reads (UMI > 0) were retained. Cells with less than 300 genes expressed or more than 10% counts of mitochondrial genes were excluded, as well as those labelled as doublet following aforementioned protocol.

DQ-OVA and OVA pulse assay. M0, M1 and M2-BMDMs were pulsed with DQ-OVA (Invitrogen) at 10 mg/mL or 100 mg/mL for 4 h at 37°C. Proteasome inhibitor MG-132 were added to suspension at a final concentration of 40 mM for 30 min, followed by DQ-OVA pulsed for 1 h. For OVA pulse, M0, M1 and M2-BMDMs were pulsed with OVA (endotoxin < 1 EU/mg) at 50 mg/mL at 37°C with gentle rotation for 4 h. And then, they were washed with PBS, CD4^+^ T cells (ISQAVHAAHAEINEAGR, I-A^d^) or CD8^+^ T cells (SIINFEKL, H-2K^b^) were added at a ratio of 1:10 co-cultured for 16 h. And then the cells were stained with CD4 or CD8, CD44, CD62L, CD69 and TCR-Vα2 on ice in the dark for 30 min and acquired on flow cytometry.

qPCR. Total RNAs were extracted with RNeasy Mini Kit (QIAGEN). RNA quantity and quality were assayed using a Nanodrop 2000 spectrophotometer (Thermo-Fisher). RNA samples were converted to cDNA through reverse transcription by RevertAid First Strand cDNA Synthesis Kit (Thermo Scientific^TM^). Applied Biosystems QuantStudio® 12 K Flex Real Time PCR thermocycler (Life Technologies™) and Forget-Me-Not™ qPCR Master Mix (Biotum EvaGreen®) were used. Primer: HA of PR8 (F: 5’-GGCCCAACCACAACACAAAC-3’, R: 5’-AGCCCTCCTTCTCCGTCAGC-3’). HA of Vic (F: 5’- ATGCTCTATTGGGGGACCCT-3’, R: 5’-AGCATTGCTTCCCCCATTCT-3’). GAPDH (F:5’- CATCACTGCCACCCAGAAGACTG-3’, R:5’-ATGCCAGTGAGCTTCCCGTTCAG-3’).

Statistical analysis. Data were expressed as mean ± SEM. All statistical analyses were performed by ANOVA test using GraphPad Prism software (version 8.2.2). *p *< 0.05 was considered significant.

## Supplementary Material

FigS1.tif

FigS5.tif

FigS3.tif

FigS2.tif

Macro_Arg1_M2 cluster7_genes.xls

FigS4.tif

## Data Availability

The authors declare that all relevant data supporting the findings of this study are available within the paper and its supplementary information. The authors declare that the relevant scRNA-seq data supporting the findings of this study are available from the corresponding authors and first authors upon reasonable request.
