# Supplementary figures and images for: Time-resolved scRNA-seq reveals transcription dynamics of polarized macrophages with influenza A virus infection and antigen presentation to T cells
Source: Emerg Microbes Infect. 2024 Aug 12;13(1):2387450. doi: 10.1080/22221751.2024.2387450 (PMC11370681; doi:10.1080/22221751.2024.2387450)

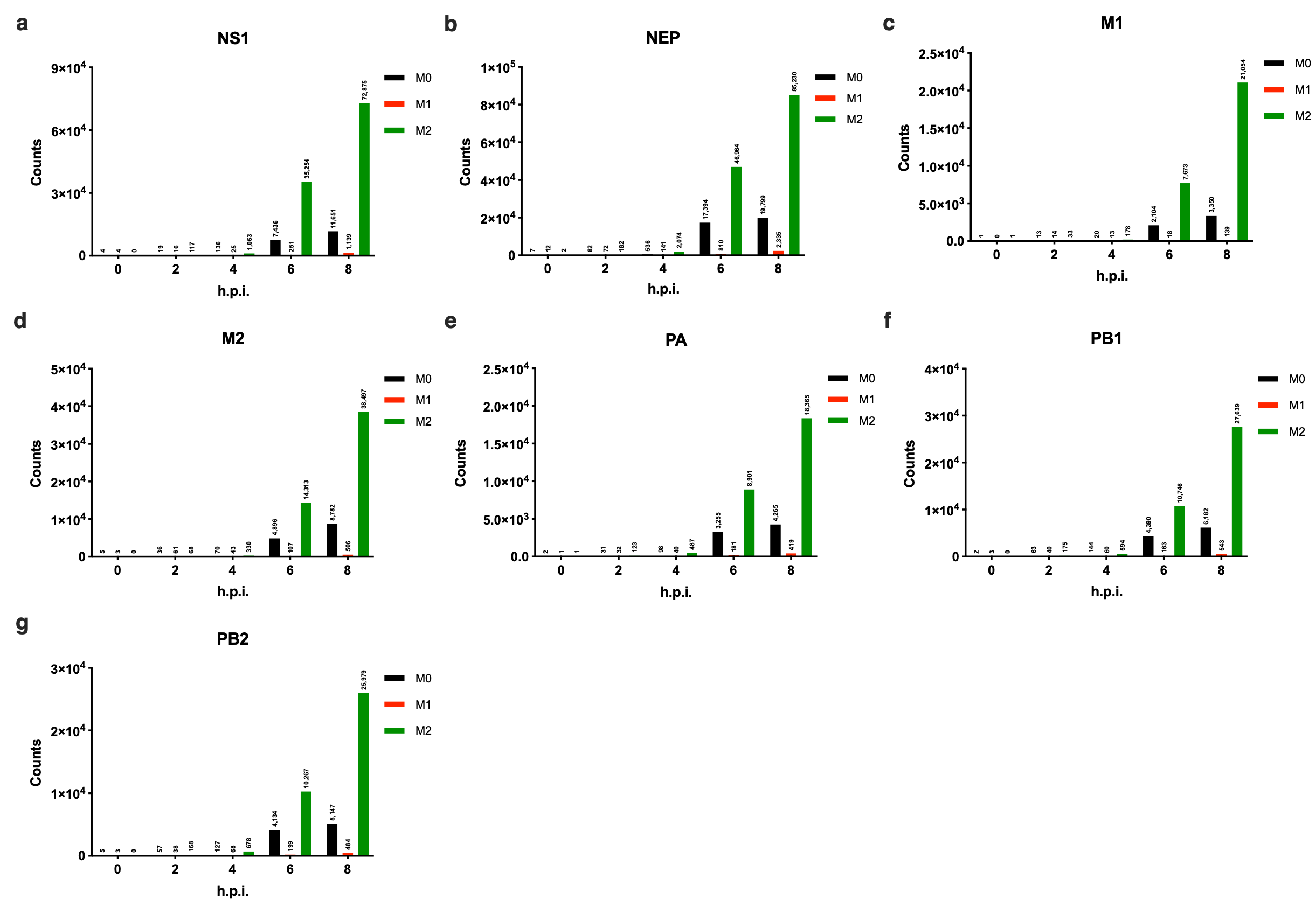

Supplement: FigS1.tif [file TEMI_A_2387450_SM6507.tif]

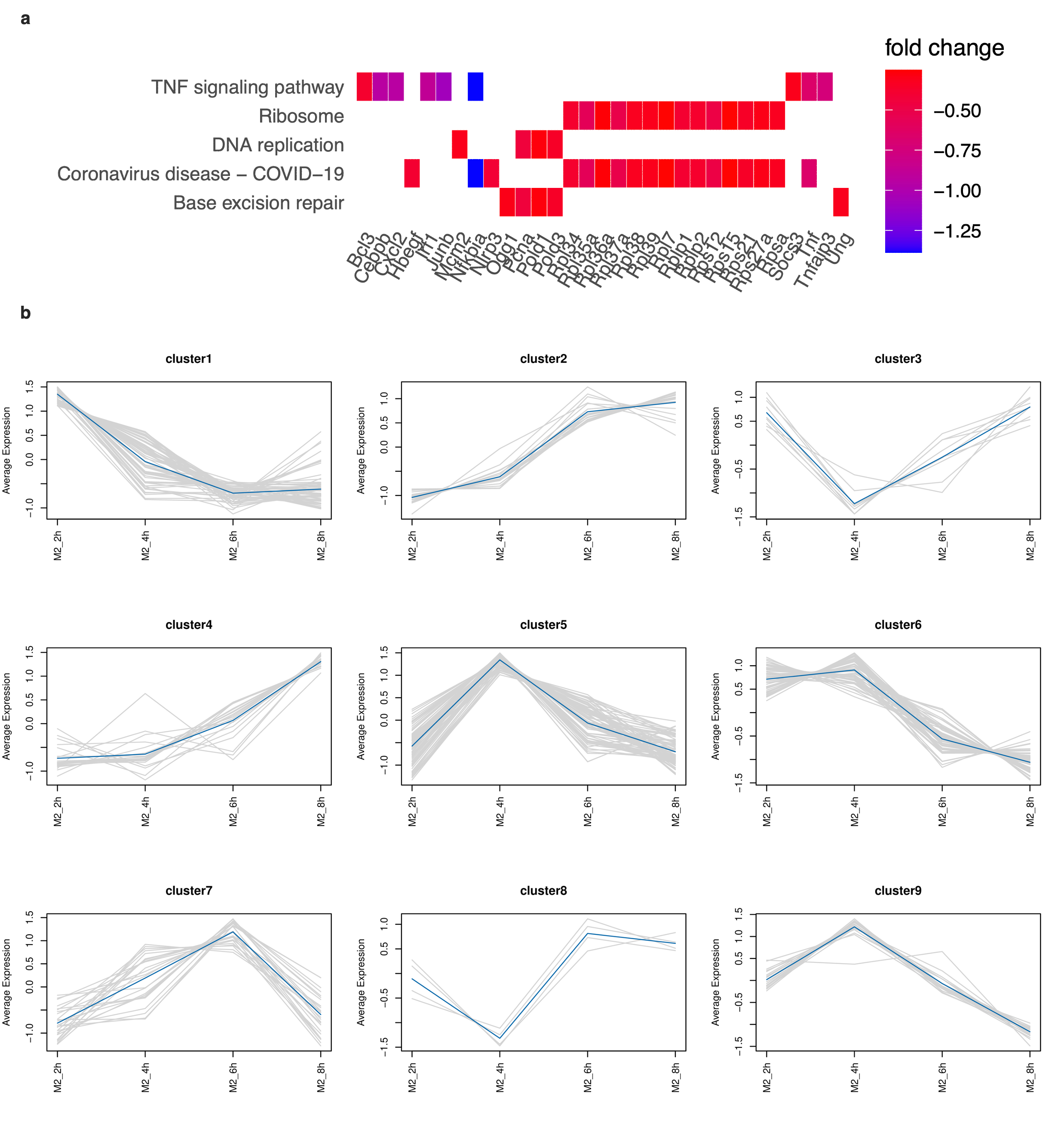

Supplement: FigS5.tif [file TEMI_A_2387450_SM6506.tif]

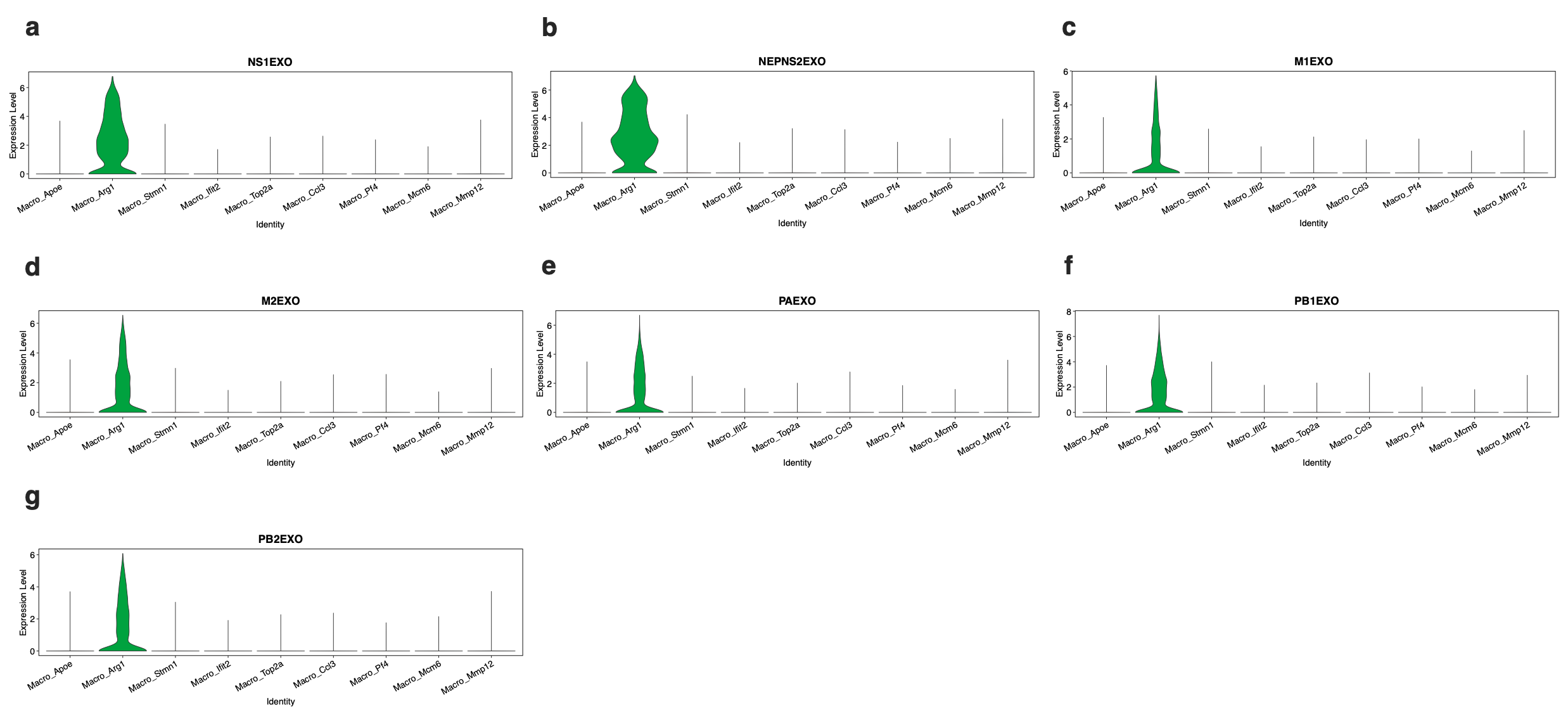

Supplement: FigS3.tif [file TEMI_A_2387450_SM6505.tif]

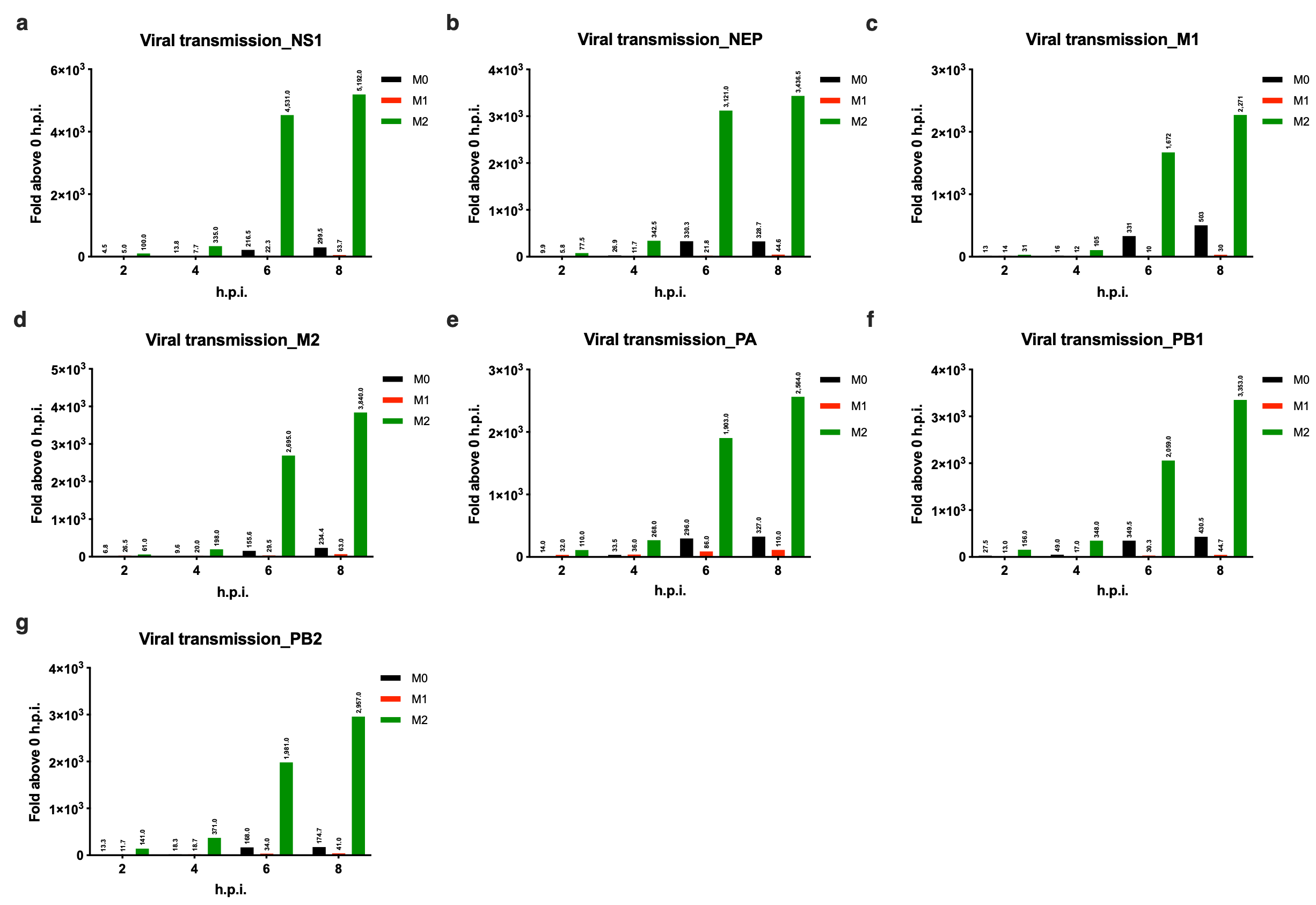

Supplement: FigS2.tif [file TEMI_A_2387450_SM6504.tif]

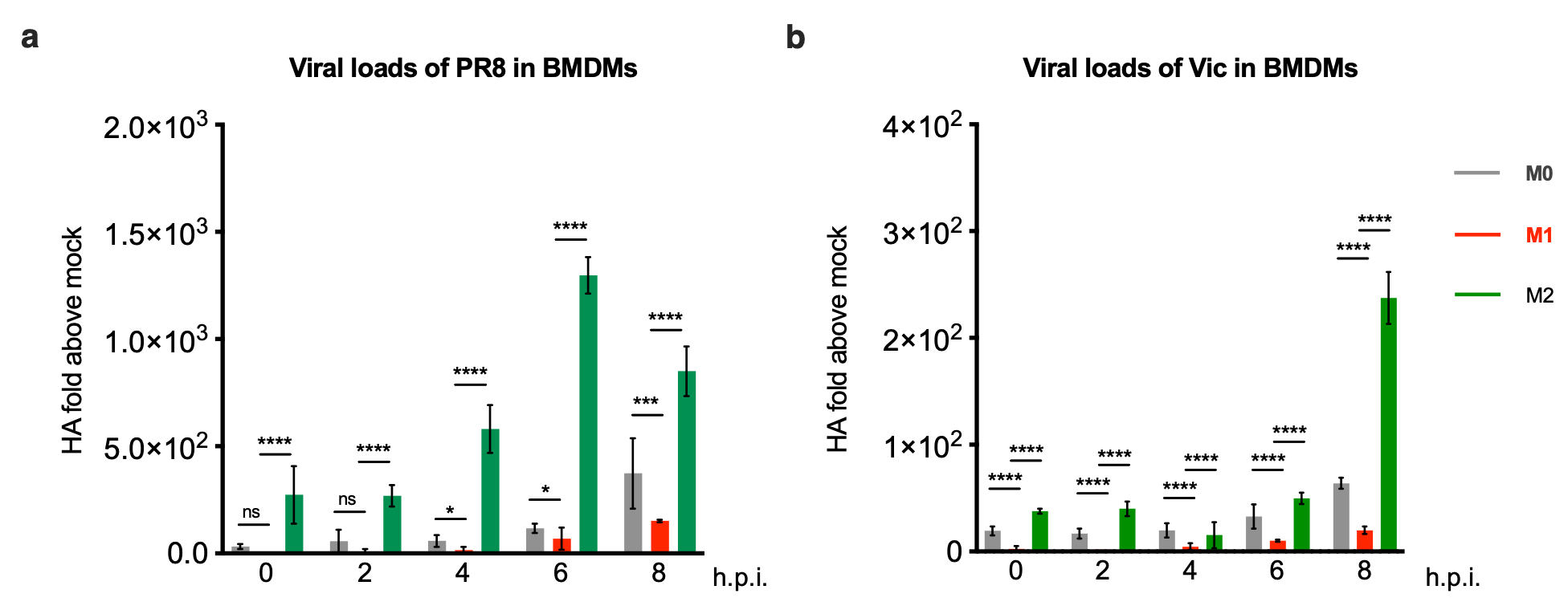

Supplement: FigS4.tif [file TEMI_A_2387450_SM6502.tif]
